# Genetic guidelines for translocations: Maintaining intraspecific diversity in the lion (*Panthera leo*)

**DOI:** 10.1111/eva.13318

**Published:** 2021-12-06

**Authors:** Laura D. Bertola, Susan M. Miller, Vivienne L. Williams, Vincent N. Naude, Peter Coals, Simon G. Dures, Philipp Henschel, Monica Chege, Etotépé A. Sogbohossou, Arame Ndiaye, Martial Kiki, Angela Gaylard, Dennis K. Ikanda, Matthew S. Becker, Peter Lindsey

**Affiliations:** ^1^ Department of Biology University of Copenhagen Copenhagen Denmark; ^2^ City College of New York New York New York USA; ^3^ FitzPatrick Institute of African Ornithology DSI‐NRF Centre of Excellence University of Cape Town Cape Town South Africa; ^4^ Institute for Communities and Wildlife in Africa University of Cape Town Cape Town South Africa; ^5^ School of Animal, Plant and Environmental Sciences University of the Witwatersrand Johannesburg South Africa; ^6^ Wildlife Conservation Research Unit University of Oxford Oxford UK; ^7^ TRACE Forensic Network Edinburgh UK; ^8^ Panthera New York New York USA; ^9^ Institute of Environmental Sciences (CML) Leiden University Leiden The Netherlands; ^10^ Kenya Wildlife Service Nairobi Kenya; ^11^ Laboratoire d’Ecologie Appliquée Université d’Abomey‐Calavi Cotonou Benin; ^12^ Laboratoire de BIOPASS Dakar Sénégal; ^13^ Département de Génie de l’Environnement Université d’Abomey‐Calavi Cotonou Benin; ^14^ Conservation Development & Assurance Department African Parks Network Johannesburg South Africa; ^15^ Tanzania Wildlife Research Institute (TAWIRI) Arusha Tanzania; ^16^ Zambian Carnivore Programme Mfuwe Zambia; ^17^ Department of Zoology and Entomology Mammal Research Institute University of Pretoria Pretoria South Africa; ^18^ Environmental Futures Research Institute Griffith University Nathan Queensland Australia; ^19^ Wildlife Conservation Network San Francisco California USA

**Keywords:** augmentation, captive, CITES, genetic variation, reintroduction, trade

## Abstract

Conservation translocations have become an important management tool, particularly for large wildlife species such as the lion (*Panthera leo*). When planning translocations, the genetic background of populations needs to be taken into account; failure to do so risks disrupting existing patterns of genetic variation, ultimately leading to genetic homogenization, and thereby reducing resilience and adaptability of the species. We urge wildlife managers to include knowledge of the genetic background of source/target populations, as well as species‐wide patterns, in any management intervention. We present a hierarchical decision‐making tool in which we list 132 lion populations/lion conservation units and provide information on genetic assignment, uncertainty and suitability for translocation for each source/target combination. By including four levels of suitability, from ‘first choice’ to ‘no option’, we provide managers with a range of options. To illustrate the extent of international trade of lions, and the potential disruption of natural patterns of intraspecific diversity, we mined the CITES Trade Database for estimated trade quantities of live individuals imported into lion range states during the past 4 decades. We identified 1056 recorded individuals with a potential risk of interbreeding with wild lions, 772 being captive‐sourced. Scoring each of the records with our decision‐making tool illustrates that only 7% of the translocated individuals were ‘first choice’ and 73% were ‘no option’. We acknowledge that other, nongenetic factors are important in the decision‐making process, and hence a pragmatic approach is needed. A framework in which source/target populations are scored based on suitability is not only relevant to lion, but also to other species of wildlife that are frequently translocated. We hope that the presented overview supports managers to include genetics in future management decisions and contributes towards conservation of the lion in its full diversity.

## 1 INTRODUCTION

Translocations of large mammals, such as the lion (*Panthera leo*), are an increasingly important conservation management tool (Berger‐Tal et al., 2020; Briers‐Louw et al., 2019; Hunter et al., 2007; Seddon, 2010; Trinkel et al., 2008). Currently, information on the genetic background is rarely taken into account when managers select source and target populations (Laikre et al., 2010). Patterns of intraspecific diversity reflect the species´ evolutionary history (Bertola et al., 2016) and contain the evolutionary potential, encompassing adaptability for possible future changes in the environment (Lande & Shannon, 1996; Mitchell‐Olds et al., 2007). Contrary to the notion that mixing between populations increases diversity, large‐scale human‐mediated mixing may lead to homogenisation (Gippoliti, Cotterill, Groves, et al., 2018; Gippoliti, Cotterill, Zinner, et al., 2018; Olden et al., 2004). Translocations that ignore the distribution of intraspecific genetic variation may not only disrupt and erode existing patterns of variation, but also catalyse direct adverse effects, e.g., individuals may not be well adapted to the climate or pathogen load of the new environment, leading to high mortality and overall limited success of the management intervention (Banes et al., 2016; Bellis et al., 2020). Intraspecific diversity is crucial to conserve, as it increases the chances of long‐term survival for populations and ultimately species (Frankham, 2005; Reed & Frankham, 2003). To avoid further loss of intraspecific variation and to maximize the chance of translocation success, it is essential to integrate knowledge on genetic variation into the decision‐making process.

Here, we use the lion as a case study, as it is a frequently translocated flagship species, has a broad distribution spanning two continents, and well‐known phylogeographic patterns that are mirrored by several other savannah species (Bertola et al., 2016). In the past few decades, lions have undergone a drastic decline in their habitat and population numbers (Bauer et al., 2015; Riggio et al., 2013). The current number of free‐roaming lions is estimated to be 23,000–39,000 (Bauer et al., 2016), with populations in Africa restricted to 18%–25% of the savannah area (Riggio et al., 2013). This is indicative of a broader decreasing trend in wildlife (Ceballos et al., 2017; Craigie et al., 2010). Wildlife, and particularly large carnivores, are increasingly confined to fragmented protected areas with limited connectivity (Newmark, 2008; Wegmann et al., 2014).

Reasons for lion translocations vary. When focussing on the release site, it may be to (1) reintroduce individuals in areas where lions have become locally extinct (henceforth ‘reintroduction’; see Box 1) or (2) introduce into an existing population with the aim of increasing diversity or boosting population growth (henceforth ‘augmentation’; see Box 1). The decision to translocate an animal may also be driven by a local problem at the source site, such as local overpopulation and/or unsustainable impacts on prey populations (Miller & Funston, 2014). It may be a way of nonlethal control of a ‘damage causing animal’, which is moved away from the area where it causes conflict, e.g., by raiding cattle. In addition to these management interventions, which are often executed by or with the support of governmental organisations, there are also translocations partly driven by more personal and financial objectives, such as establishing a game reserve, hunting concession, zoo or private collection. Here, we argue that regardless of the reason for translocation, if there is a chance of (future) interbreeding with free‐roaming lion populations, the genetic background of both the source and the target population need to be taken into account during the decision‐making process. This is of particular importance for interventions that span a larger geographic scale, since there is an increased risk that selected populations will be from differentiated genetic clades. Even though within‐country translocations are a common intervention to resolve human‐wildlife conflict, we focus on long‐distance translocations and the accompanying genetic risks.

BOX 1 **Definitions of reintroduction, augmentation, assisted colonisation and ecological replacement**

**Table:** 

Several sources provide detailed definitions of the various types of translocation interventions (IUCN, 2013; Seddon, 2010). We outline how we use the different terms below: Within the species’ range: if conspecifics are absent (i.e., locally extirpated): **reintroduction** (synonym: reestablishment). if conspecifics are present: **augmentation** (synonyms: population reinforcement, supplementation, restocking, enhancement and assisted gene flow). If the goal is specifically to increase population fitness by the introduction of new alleles, this may be referred to as **genetic rescue**. Outside of the species’ range (not common for lions): if the purpose is to avoid extinction as a result of loss of populations within the species’ range: **assisted colonisation** (synonyms: assisted migration and managed relocation). if the purpose is to restore ecological function, e.g., as a result of the local extinction of an ecologically similar species: **ecological replacement** (synonyms: ecological substitute and taxon substitution). If the focus is on the translocated individual, rather than on the target population or the ecological function it may provide, some organisations use the term **rewilding**. This typically involves a captive animal being introduced into an area where it can be free‐roaming for nonconsumptive purposes and possibly lead to restoring populations and ecological function (Carey, 2016).

In the case of lions, of the categories mentioned in Box 1, reintroductions and population augmentations are most common, and we therefore focus on these when we refer to translocations. We emphasise that the classification depends on the context in which a translocated individual is being released. For example, translocation as nonlethal control, such as the translocation of a damage causing animal, could technically fall within any of the main categories, depending on the target location for release. Furthermore, there are major differences among countries in how wildlife is managed, including what is regarded as a wild population. In the Republic of South Africa (RSA), lions and other wildlife are most intensively managed, often in fenced reserves (Miller et al., 2013; Wells, 1996). These intensively managed populations have a somewhat exceptional status, since they are the result of reintroductions with subsequent regular translocations between populations to mimic natural movements (Miller et al., 2015). They are located in at least 45 smaller, fenced areas (<1000 km^2^), harbouring ~700 lions (Miller et al., 2013). We include them in this study as ‘managed metapopulation’ (unlike the lions from the captive breeding facilities, which are not included in this study), as they are frequently used as a source for translocations (African Parks, 2015; Briers‐Louw et al., 2019; also see the CITES Trade Database mining exercise in this study). In many cases, the genetic background is known (Miller et al., 2015), but this type of information is often too scattered or inaccessible for managers to be included in conservation decisions. Hence, there is a need for a thorough overview of the current state of knowledge on lion genetic variation and the translation of this genetic information into a decision‐making tool for managers. Scattered data, as well as poor documentation of previous translocation efforts, have also been identified as a management challenge for other species, such as black rhinoceros (Moodley et al., 2017), giraffe (Muller, 2019; Winter et al., 2019) and wildebeest (Grobler et al., 2011). Considering the similarity in phylogeographic patterns across African mammals (Bertola et al., 2016), well‐designed recommendations for lions may also indirectly provide guidelines for other species.

**FIGURE 1:**
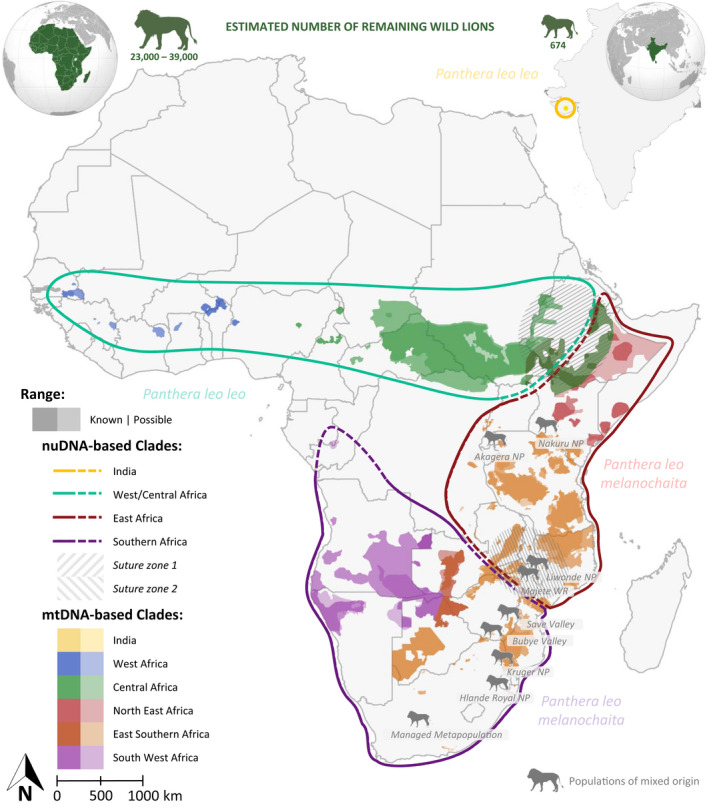
The distribution of genetic variation in the lion, based on previous studies (see text). Colours of the lion range indicate genetic lineages based on mitochondrial DNA; delineation indicates genetic lineages based on nuclear DNA. Natural suture zones are indicated by shading. Dashed lines indicate uncertainty regarding the exact boundary, as this is inferred from available sampling localities and/or suture zones. This also holds true for the overlapping colours in the lion range in southern Africa. We indicate the availability of genetic data and the certainty of these inferences in Table S1. Lion populations affected by previous translocations crossing phylogenetic clades leading to a hybrid character are indicated with grey lion symbols (details in Table S1)

During the past 10 years, our understanding of intraspecific patterns of variation in the lion has substantially improved (Antunes et al., 2008; Barnett et al., 2014; Barnett et al., 2006; Bertola et al., 2019; Bertola et al., 2015, 2016; Bertola, van Hooft, et al., 2011; Curry et al., 2020; de Manuel et al., 2020; Dubach et al., 2005; 2013; Tensen et al., 2018) (Figure 1). Here, we refer to genetic variation for both differentiation between populations or genetic clades, and diversity within populations, both of which can be affected by translocations. Patterns of differentiation between populations are influenced both by nonadaptive drivers, including demographic histories and connectivity across the landscape, as well as potential adaptation to local conditions (Cortázar‐Chinarro et al., 2017; Pfeifer et al., 2018). At a larger geographic scale, species may lose variation by local extinctions of populations (Ceballos et al., 2017); at the local scale, small and isolated populations risk losing genetic diversity as a result of individuals becoming more closely related (inbreeding) and through stochastic processes (genetic drift). Translocations may counteract both by reintroducing individuals to areas where they have previously gone extinct and by mimicking natural gene flow to counteract local loss of genetic diversity and associated fitness consequences (inbreeding depression) (Gaitán‐Espitia & Hobday, 2021).

As global biodiversity is declining at an unprecedented rate, it is key to assess patterns of genetic variation, both between and within populations, and incorporate relevant data into population management and policy (Des Roches et al., 2021; Hoban et al., 2020; Laikre et al., 2020). Here we formulate recommendations on including genetic information as part of the decision‐making process for translocations. Natural dispersal capability of lions can provide guidance; however, existing patterns of genetic variation should be taken into account, in particular for long‐distance translocations. This information can be used to guide decisions for source/target populations for translocations, as well as to review past management interventions. To gain insight into the extent and the directions of past translocations, we collected information from the CITES Trade Database on the transboundary trade of lions within and into Africa, spanning four decades. We focus on cases where there is a considerable risk that translocated animals have, or may in the future, interact and breed with wild lions in the target area, since this has the potential to compromise existing patterns of genetic variation.

We acknowledge that there are constraints related to the lack of availability of suitable genetic stock and/or difficulties associated with getting permission for translocations from certain countries, which may result in the reintroduction of genetically suboptimal individuals to target sites. This study, however, was not executed with the goal to criticise past efforts or restrict current initiatives, but rather to understand the magnitude and direction of lion translocations and to provide a resource for future planning. In order to select populations, individuals and release sites in line with our current understanding of lion genetic variation, we therefore developed a hierarchical decision‐making tool, and we highlight different levels of suitability, which can be used by decision makers in lion range states when planning translocations.

## 2 METHODS

### 2.1 Phylogeographic patterns

To interpret recent translocations and ongoing translocation plans in the context of known patterns of intraspecific variation, we created a list of 132 lion conservation units (LCUs) (IUCN SSC Cat Specialist Group, 2006a, 2006b; Riggio et al., 2013) and assigned each LCU to a phylogenetic clade (Table S1). For completeness sake, we incorporated transboundary populations. We include small managed reserves in RSA, referred to hereafter as the ‘managed metapopulation’; however, we exclude lions from captive breeding facilities. Also included is the recently identified population in Gabon (Batéké Plateau) (previously thought to be extinct) and newly restored populations in Malawi (Liwonde Ecosystem and Majete Wildlife Reserve), Rwanda (Akagera National Park [NP]) and Eswatini (Hlane Royal NP). Here, we refer to countries with current, wild lion presence as “lion range states”. We acknowledge that this definition is somewhat restrictive, resulting in countries with recent lion population extinctions losing this status. Therefore, translocations to some nonrange states can still be within the historical, natural range of the lion.

For assignment of each population to a (putative) genetic clade, we make a distinction between nuclear DNA (nuDNA, based on microsatellite and/or single nucleotide polymorphisms [SNPs]) and mitochondrial DNA (mtDNA) patterns, as observed in previous studies. Although these patterns are largely congruent, in certain parts of the lion range, there are differences in patterns derived from these two types of genetic markers, which can be explained by local demographic histories and migration patterns (Bertola, 2015) (Figure 1). Since not all LCUs have been included in previous genotyping efforts, for each designation, we included an assessment of certainty (high, fairly high, medium) and associated data sources. We stress that the delineation of the clades is based on available data (i.e., sampling localities), and increase in sampling may allow for a more precise indication of boundaries between genetic clades in the future. The same holds true for the indicated suture zones, where different genetic clades naturally overlap and where individuals have been identified to show hybridization between clades.

Based on the phylogenetic clade assignments of LCUs and associated certainty (Table S1), we generated a table with suggested source populations for each of the LCUs (Table S2). Here, we include a first choice, second choice and third choice (the latter only available for *P. l. melanochaita*) as a pragmatic and hierarchical approach for finding suitable source populations for conservation translocations. We define the ranking roughly as follows: (1) first choice: source and target populations fall within the same nuDNA and same mtDNA clade; (2) second choice: source and target populations fall within the same nuDNA clade but are from differentiated mtDNA clades; (3) third choice: source and target populations are from differentiated nuDNA clades, but from the same subspecies. We also include ‘no option’, which represents the introduction of the alternative subspecies or individuals of unknown genetic origin, which we strongly advise avoiding. We also categorize a translocation from the same subspecies from West or Central Africa into India (or vice versa) as ‘no option’ because of the strongly differentiated character of the Indian population. This categorization is in line with widely accepted recommendations, often summarized as keeping populations separated if there is no evidence of recent gene flow (Frankham et al., 2017; Liddell et al., 2021; Ralls et al., 2018).

A complete overview of source/target combinations for translocations is shown as a full matrix in Table S3. In addition to the four levels of suitability, indicated as dark green, light green, yellow and red, we highlight the populations, which have been affected by human‐mediated hybridization as a result of previous translocations as faded colours (faded yellow and faded orange). These populations, also highlighted in Figure 1, are in principle not suited as a source for future translocations; however, hybridization is likely not represented across all individuals equally. Hence, if there is the opportunity for genetic analyses of candidates for translocations, these populations may be considered as a source.

### 2.2 CITES trade database

CITES (the Convention on International Trade in Endangered Species of Wild Fauna and Flora) is a multilateral legally binding treaty covering the transboundary trade of >35,000 species of plants and animals, including lions. Parties to CITES are obliged to compile and submit annual reports on their international trade in species listed on Appendix S1 to the Secretariat (CITES, 2013), which are entered into the online CITES Trade Database maintained by UNEP‐WCMC (https://trade.cites.org/). To review the magnitude and direction of past international transport of lions, statistics available for legal live lion exports/imports were mined from the online Trade Database for the period 1983—2019 (years for which information is present; no data pre‐1983 met our criteria). We stress that the numbers retrieved from the database merely reflect the intention of trade and will thus deviate from the number of actual translocated individuals. Details of our approach and the definitions of source and purpose codes, as captured in the CITES Trade Database, can be found in Appendix S1.

The available CITES trade data were amalgamated into four categories for analysis, namely the numbers of lions listed on consolidated permits that were (i) wild lions (source codes ‘W’, ‘F’ and ‘R’, respectively wild, born in captivity (but do not fulfil the definition ‘bred in captivity’, e.g., F1), and ranched), intended for (re)introduction into the wild (purpose code ‘N’); (ii) captive lions (source code ‘C’), intended for (re)introduction into the wild (purpose code ‘N’); (iii) wild lions (source codes ‘W’, ‘F’ and ‘R’), to be exported with the intention of commercial trade, breeding in captivity, and trophy hunting purposes (purpose codes ‘T’, ‘B’ and ‘H’) and (iv) captive lions (source code ‘C’), to be exported with the intention of commercial trade, breeding in captivity and trophy hunting purposes (purpose codes ‘T’, ‘B’ and ‘H’) (Table S4: CITES data original). Since the information on the CITES Trade Database is not based on the actual numbers of lions traded but is instead based on the quantities listed on the export/import permits issued by the respective countries, the consolidated country information presented in this paper shows the maximum numbers of lions that were intended to be translocated when the CITES permits were issued. An unknown proportion of these lions were not exported. Nevertheless, these data inform the relative scale of transboundary lion translocations between countries, and the purposes and origins thereof. We speculate that there are also occasions where live lions are being translocated without the proper documentation, which are therefore not documented in the CITES Trade Database (Williams et al., 2015).

Based on scientific and grey literature and expert knowledge of authors, it became clear that, following our strict rules as outlined in Appendix S1, several translocation events had been misclassified. This mostly applied to translocations from RSA, which was the major contributor to cross‐boundary lion trade. For example, certain lions in the managed metapopulation in RSA were classified as ‘captive’ while others were classified as ‘wild’ (see ‘Discussion’). The genetic background of lions in the managed metapopulation is relatively well understood (Miller et al., 2015) and thus can be classified within our scale, while that of the captive‐bred lions is not and should remain as ‘no option'. Thus, we separated these sources: captive‐bred lions were left as captive, while lions in the managed metapopulation were classified as ‘Wild 2’ or ‘W2’. Export permits with ‘F’ or ‘R’ as the classification were also classified as ‘W2’. In cases where no source code was listed on export records, we were able to infer some information based on the years the permits were issued. For example, small managed reserves in the managed metapopulation of RSA were only established starting in 1991 (Funston, 2008; Slotow & Hunter, 2009), and very few captive facilities existed pre‐1994 (Williams & ‘t Sas‐Rolfes, 2019); thus, movements out of RSA before the mid‐1990s were most likely to be wild‐sourced if the source information is missing. Translocations out of RSA after 1999 were very unlikely to have been from Kruger NP due to bovine tuberculosis spreading to lions (Slotow & Hunter, 2009). Translocations from the RSA side of the Kgalagadi Transfrontier Park have become increasingly rare in recent years. Thus, any permits stating ‘W’ as the source code post‐2005 were assumed to be from the managed metapopulation and classified as ‘W2’. Numbers and notes for each of the categories are shown in Table S5 (CITES data adjusted).

## 3 RESULTS

### 3.1 Phylogeographic patterns

**FIGURE 2:**
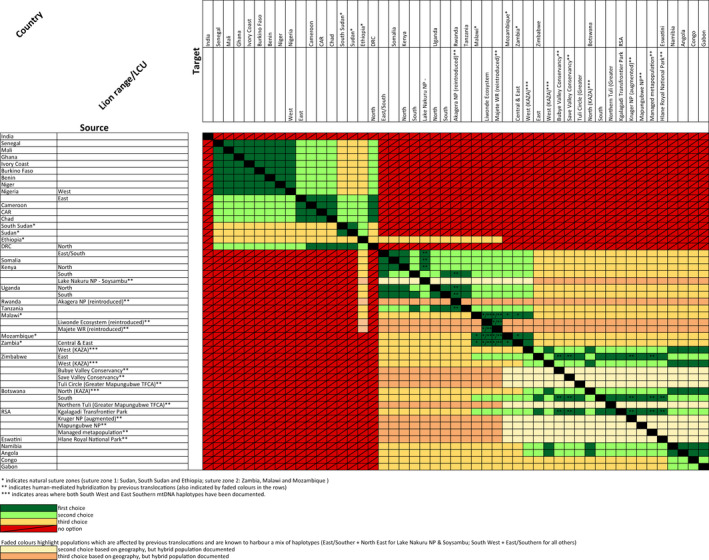
Matrix of lion range countries, indicating the suitability of each source/target combination for conservation translocations. Suitability is indicated by colours: dark green = ‘first choice’, light green = ‘second choice’, yellow = ‘third choice’ and red = ‘no option’. * indicated natural suture zones, ** indicate human‐mediated suture zones, also indicated by faded colours in their suitability scoring. Table S3 provides a more detailed matrix, listing all lion ranges/lion conservation units (LCUs)

**FIGURE 3:**
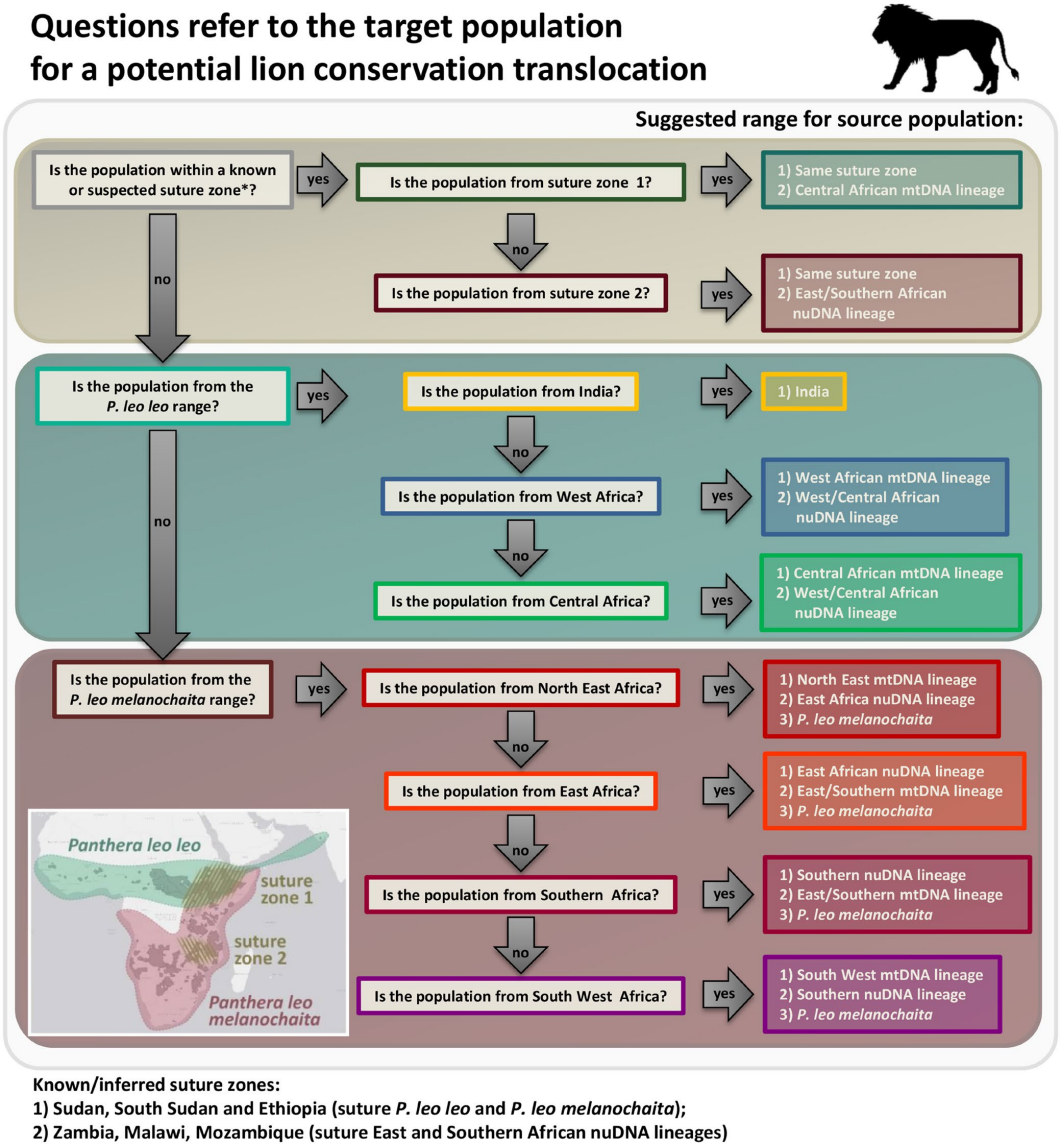
Decision tree to guide the choice of suitable source population for lion conservation translocations. Note that this decision tree does not include populations with a hybrid character due to previous translocations (grey lion symbols Figure 1, faded colours Figure 2 and Table S3)

Based on the phylogenetic assignment, associated certainty (Table S1) and suggested suitability for potential source populations (Table S2), available options and best practice decisions regarding the selection of a source/target population are summarized in a full matrix of source/target population pairs (Figure 2 depicts a summary per country, Table S3 contains the full matrix of all LCUs) and a decision tree (Figure 3). Two natural suture zones where different nuclear clades overlap are highlighted: suture zone 1: Sudan, South Sudan and Ethiopia, and suture zone 2: Zambia, Malawi and Mozambique. In addition to these, in the Kavango Zambezi (KAZA) Transfrontier Conservation Area diverged mtDNA clades overlap, while all populations are assigned to the Southern nuDNA clade. Based on the available data in the literature (Antunes et al., 2008; Bertola et al., 2016; Curry et al., 2015; Dures et al., 2019), we postulate that this overlap extends into the Zimbabwean and Zambian part of KAZA, as well as part of Botswana (indicated in mixed colours in Figure 1). Because this overlap of mtDNA clades is not apparent in nuDNA data, we do not regard this as a suture zone. We also highlight human‐mediated hybridization as a result of the introduction of lions from Etosha NP (Namibia, South West mtDNA haplotype) into the Kruger NP area (RSA, East/Southern mtDNA haplotype) and potentially hybrid lions from RSA into Zimbabwe, Eswatini, Rwanda and Malawi, indicated with grey lion symbols on the map (Figure 1). A second case in which human‐mediated hybridization has likely changed natural patterns of diversity is in Lake Nakuru NP‐Soysambu (Kenya) (East/Southern mtDNA haplotype), where four out of six founder individuals came from Aberdares NP (North East mtDNA haplotype), also indicated with a grey lion symbol. Note that these potentially hybrid individuals as a result of previous translocations are not explicitly included in the decision tree (Figure 3).

It must be noted that around natural suture zones and in the case of potential hybrid populations, the matrix is not symmetrical: movement into suture zones (of lions from one of the overlapping clades) was scored as more favourable than movement from suture zones (where possible hybrid individuals exist). We scored the admixed populations resulting from past translocations (as indicated on Figure 1) as in principle unsuitable as a source, highlighted by faded colours in Figure 2 and Table S3. However, additional data could be helpful to identify the genetic background of individuals for future translocations.

### 3.2 CITES trade database

To assess the degree to which past translocations may have influenced existing patterns of genetic variation, we evaluated records of 1056 individuals from the CITES Trade Database (Table S4: CITES data original). By using original source data from the Database, 848 (81%) were captive‐sourced (including 290 individuals from outside lion range states, Table S6), and hence, we categorised them as unsuitable candidates, given that their genetic background is often unknown. We acknowledge that this may be restrictive, and under certain circumstances, an individual that technically falls within the captive category may be genetically suitable for reintroduction purposes. Therefore, we re‐examined the data, including scientific literature, grey literature and personal communications, which resulted in the addition of the ‘W2’ category (Table S5). To avoid confounding these individuals with true wild (‘W1’) individuals, and to avoid confusion with the standard suitability score, we marked them with the colour blue. It is known that some of these individuals were sourced from populations harbouring hybrid individuals as a result of previous translocations. In this updated overview, including the ‘W2’ category, the number of captive individuals is reduced to 772 (73%), which includes 290 captive lions from outside current lion range states (and two of unknown origin), leaving a total of 480 lions (45%) as captive from lion range states.

**FIGURE 4:**
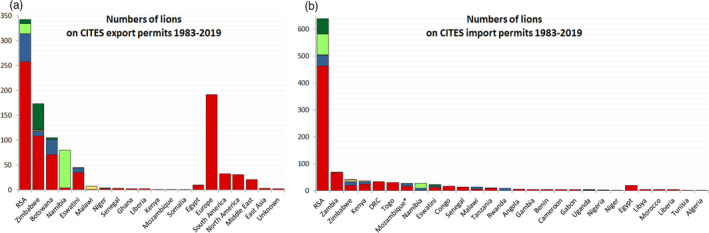
Barplots indicate the number of lions for which permits have been documented in the CITES Trade Database, split up per exporting (a) and importing (b) country. The colours indicate which proportion of the trade falls in each of the suitability categories: dark green = ‘first choice’, light green = ‘second choice’, yellow = ‘third choice’, red = ‘no option’ and blue for individuals from the ´W2´ category

**FIGURE 5:**
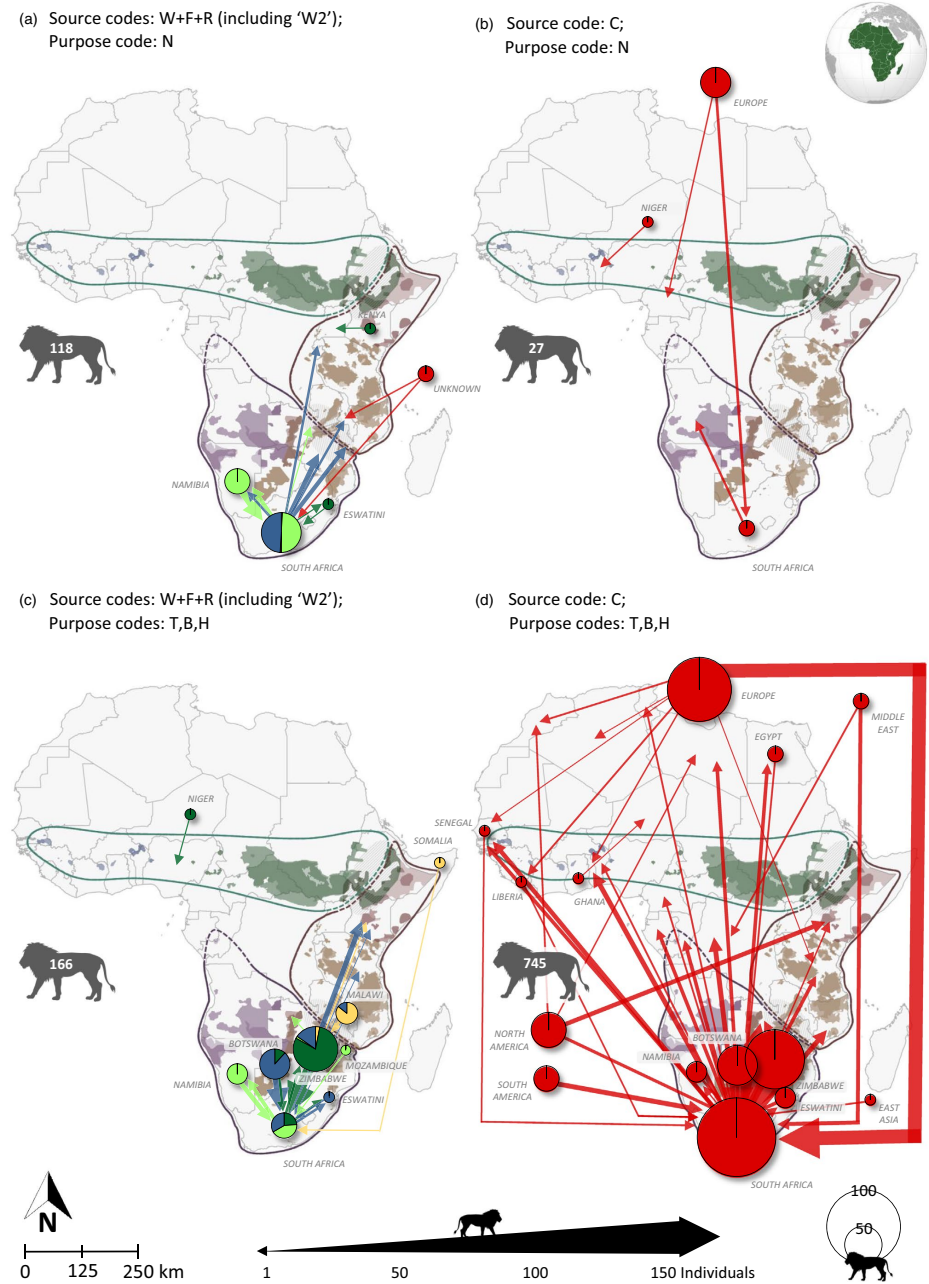
Maps showing international lion trade using CITES data adjusted (Table S5), split up in the four trade categories depending on source and purpose codes and including category ‘W2’. The width of the arrow reflects the number of lions on the permits. The colours indicate the suitability category for each of the source/target combinations: dark green = ‘first choice’, light green = ‘second choice’, yellow = ‘third choice’ and red = ‘no option’. ´W2´ individuals are included as blue arrows. Circle size and arrow width indicate the number of translocated lions

Focussing on the origin of the individuals for which CITES records were obtained, and using Table S5 (including the ‘W2’ category), RSA was the main exporter with 342 (32%) of all individuals (Figure 4a). Zimbabwe, Botswana and Namibia also contributed considerably, with 16%, 10% and 8%, respectively. RSA was also the main importer, with 638 (60%) of all imported individuals, and Zambia (7%) in second place (Figure 4b). Using the suitability score, 6% of all individuals fall in the ‘first choice’ category, 9% ‘second choice’, 1% ‘third choice’ and 10% ‘W2’. Despite the addition of the ‘W2’ category, 73% (*N* = 772) of the individuals included in the CITES Trade Database would still receive a ‘no option’ recommendation. The vast majority of the individuals in this category receive this recommendation due to their captive origin. We summarize these results, both for CITES data only and for CITES data adjusted in Table S6. If we were to assume that the country of origin is representative of the genetic clade, even for the captive individuals, 383–413 individuals would still be identified as ‘no option’ (depending on the DRC release site). To illustrate the trade of live lions included in the CITES Trade Database in a geographic context, these permitted translocations were mapped for each of the four trade categories (‘W2’ translocations indicated in blue) depending on their source and purpose codes (Figure 5).

## 4 DISCUSSION

Here we present an overview of LCUs and their association to genetic clades, based on the best available evidence. We connect this to recommendations for future conservation translocations, by providing a hierarchical approach, which includes a ‘first choice’, ‘second choice’, ‘third choice’ and ‘no option’ (Figure 2, Table S3) and a decision‐making tree (Figure 3). We do not aim to reiterate overall genetic considerations for translocations, as there are several studies which cover these (Mijangos et al., 2015; Pacioni et al., 2019) but rather focus on a practical approach for lion conservation, which will hopefully support managers in their decision‐making process. As several African savannah mammals show congruent patterns in their phylogeography (Bertola et al., 2016; Fennessy et al., 2016; Lorenzen et al., 2012), the considerations presented here may also be applicable to other species.

### 4.1 Translocations: past and present

Despite people being involved in moving wildlife, including lions, throughout history (Belozerskaya, 2009; Hughes, 2003; Kalof, 2007), there is no indication that contemporary phylogeographic patterns are the result of anthropogenic disturbance. It is likely that many of these human‐mediated movements took place over a relatively short distance, and typically, the translocated individuals would end up in zoos or private collections and were unlikely to introgress into a resident population (if present). For lions, it was suggested that the Indian population may have had some influx from Africa through human‐mediated dispersal (Packer, 2013; Thapar, 2013); however, there are no genetic data, which support this claim. Another study suggests that, historically, lion populations across Africa were close to panmictic with no pronounced population structure (Curry et al., 2020). However, we postulate that given the estimated divergence times and the lack of an isolation‐by‐distance signal, the contemporary patterns in lion diversity cannot be entirely explained by recent habitat fragmentation (Bertola et al., 2016); therefore, contemporary patterns of genetic diversity should be seen as ‘natural’.

The most pronounced deviation from this natural pattern of lion diversity in free ranging populations, which may be due to recent human intervention, is the introgression of a South West mtDNA haplotype in Kruger NP (RSA) (Bertola et al., 2016). More recent translocations of individuals from a human‐mediated hybrid population, e.g., to Malawi and Rwanda (African Parks, 2015; Briers‐Louw et al., 2019; Miller et al., 2015), will have resulted in a similar disruption of the natural pattern (see Table S1 and Figure 1 for affected populations). We acknowledge that some target areas in which lions have been translocated are fenced (e.g., in Malawi) and/or with no resident lion populations in close proximity (e.g., in Rwanda). However, the decision for a source population may have long‐lasting effects spanning multiple generations; we highlight these cases because the persistent risk of fence breaches will allow for interactions between translocated individuals and individuals from a local population. We therefore urge managers to use the contemporary distribution of genetic variation, with the exception of known human‐mediated disruptions (highlighted in Figure 1), to guide future conservation translocations.

### 4.2 CITES trade database

The reports entered into the CITES Trade Database allow publically accessible trade statistics and outputs to be generated, but we note the limitations as outlined in Appendix S1 (e.g., overestimation or underestimation of individuals based on the quality and completeness of annual reports submitted to CITES). As it was our aim to assess movements of lions across international borders into lion range states with a potential impact on wild lion populations, we included several source and purpose categories, consolidated into four trade categories (see ‘Methods’). The CITES Trade Database does not list information regarding the destination other than the country name. Because some countries harbour multiple genetic clades (e.g., the split between west and east Nigeria, or the split between north and south Kenya), the information from the Database in some cases was insufficient to assess suitability of source/target populations in detail. As there is often no clear distinction between a fenced reserve, private collection or zoo, restocking of a hunting zone etc., our rationale is that by including these categories, we capture those individuals with the highest risk of interaction, and potential for breeding, with wild lions.

The distinction between ‘wild’ and ‘captive’ in the source codes is not always clear in practice, which is most strongly visible in the case of RSA. Here, lion populations exist along a gradient ranging from captive, commercially bred stocks (not included in this study), to intensively managed free‐roaming populations (‘managed metapopulation’), to traditional free‐roaming (listed LCUs) (Funston & Levendal, 2015). Most of the translocated lions from the managed metapopulation in RSA were categorized as ‘captive’ (based on the information derived from the CITES permits, see Appendix S1), even though there is a clear distinction from, e.g., zoo lions (Miller et al., 2013), and they should have been classified as wild (Funston & Levendal, 2015, J. Selier pers. comm.). For example, we reclassified the lions translocated from RSA to Rwanda from ‘captive’ to ‘wild (W2)’ after inclusion of grey literature sources confirming their origin in the RSA managed metapopulation. There are likely still some misclassified data due to lack of additional information, and thus, the captive data may be inflated. This highlights the challenges associated with using the CITES Trade Database in isolation as a source of data for conservation assessments.

The managed metapopulation in RSA has been stocked with individuals from Kruger NP, Kgalagadi Transfrontier Park and Etosha NP (Namibia), and it mostly represents a mix of two mitochondrial haplogroups (East/Southern and South West) (Miller et al., 2015). Several translocations have sourced their lions from the RSA‐managed metapopulation, including the translocations to Malawi and Rwanda, leading to these populations also representing human‐mediated hybrid lions (indicated with lion symbols in Figure 1; faded colours in Figure 2 and Table S3; and blue for all individuals in the ‘W2’ category in Figure 5). We do not claim that there is no conservation value to these individuals, and we acknowledge that despite the hybrid character of the population, this may not be equally represented across individuals. Furthermore, as the managed metapopulation in RSA is actively managed, its genetic background is not constant, making it challenging to give it a concrete translocation recommendation. Thus, we have highlighted these lions in our assessment, and additional care must be taken to determine the suitability of individual lions in relation to the intended destination. Genetic testing is becoming increasingly accessible, both in terms of technology, such as the developments of handheld devices, and in terms of lion‐specific resources, such as lion‐specific SNP panels or microsatellites (Bertola et al., 2019; Curry & Derr, 2019; Miller et al., 2014; Smitz et al., 2018). The Lion Management Forum of South Africa (LiMF; limf.co.za) can be consulted for the most recent information on these populations and combined with genetic testing to determine their suitability for translocation.

Our scoring of ‘no option’ for all captive lions mainly reflects that the genetic background of the captive population is mostly unknown (Bertola, Vrieling, et al., 2011). The need for stock for reintroduction or augmentation is a popular argument for some organisations justifying breeding lions in captivity; however, these programmes have a limited capacity to contribute to in situ lion conservation (Hunter et al., 2013). Apart from the fact that captive populations are prone to inbreeding and may have been artificially selected for certain phenotypic traits, other, nongenetic factors, such as behaviour, need to be taken into account when considering individuals for release, generally making captive lions poor candidates. However, coordinated efforts from established zoos, e.g., associated with the World Association of Zoos and Aquaria (WAZA), may play a role in safeguarding lion genetic variation in their ex situ collection, even without concrete opportunities for reintroductions or augmentations.

### 4.3 Genetic considerations

Augmentation can contribute positively to the genetic health of the resident population by two mechanisms: (i) alleviating the negative fitness effects of inbreeding depression (Tallmon et al., 2004) and (ii) increasing adaptive potential for natural selection to act upon (Aitken & Whitlock, 2013). When considering augmentation, it is advisable to make a distinction between inbreeding (low genetic diversity as a result of mating between individuals related by descent) and inbreeding depression (negative fitness effects as a result of low genetic diversity) (Frankham et al., 2017). Fitness data are often not readily available, and it may be impossible to gather these data within a reasonable timeframe and/or given available resources, considering that extinction risk rises rapidly in an inbred population (O’Grady et al., 2006). There are a few case studies dealing with genetic rescue in lions (Miller et al., 2020; Trinkel et al., 2008) and other felids (Johnson et al., 2010). Large source populations are generally seen as the most effective way to increase diversity in a genetically depauperate population (Frankham, 2015). As long as the target population remains small and isolated, continuous input from the source population may be necessary to avoid inbreeding in subsequent generations (Hedrick & Fredrickson, 2010), and sourcing from a larger, more diverse population will allow lower levels of gene flow (i.e., follow‐up reintroductions) before levels of inbreeding reach harmful levels.

Despite clear evolutionary benefits to higher genetic diversity, we note that the ‘preferred state’ of genetic composition is highly context dependent, primarily related to anthropogenic impact on observed patterns of diversity. We acknowledge differentiation between populations; however, if this is demonstrably the result of recent, human‐mediated fragmentation of the habitat, it may be advisable to counteract this by restoring connectivity, with restored gene flow leading to a decrease in differentiation (Frankham et al., 2011; Ralls et al., 2020). In general, higher heterozygosity is the preferred state; however, if human‐mediated gene flow (e.g., through translocations) leads to the outbreeding of two strongly differentiated populations, it may result in inflated heterozygosity, as well as the disruption of evolutionarily distinct clades and possibly outbreeding depression (Frankham et al., 2011; Ralls et al., 2020). This illustrates that the preferred state of populations and individuals needs to be seen in a bigger picture, including a historical context.

Although we focus here on the genetic considerations for conservation translocations, we also want to highlight the importance of ecological, behavioural and socioeconomic aspects of these interventions (IUCN, 2013). Ecological considerations may play a role for augmentations, e.g., as there may be a risk that the resident and/or the translocated individuals are exposed to new pathogens. The absence of feline immunodeficiency virus (FIV) in lions from Etosha NP (Spencer et al., 1992) was an important reason for their popularity in former translocation efforts, explaining the presence of South West haplotypes in many of the South African lion populations. Ecological considerations also play a role in reintroductions (i.e., with no resident lion population present), as biological communities can go through drastic shifts after local extinctions, even leading to community closure, which renders future reintroductions unsuccessful (Lundberg et al., 2000; Tielke et al., 2020). Removing an animal from a population and introducing it into another population may have a considerable impact on individual behaviour and social structure, especially for group living species (Goldenberg et al., 2019). In all cases, it is crucial to ensure that the original pressure leading to the decline of the target population has been removed. As human communities often live in the vicinity of release sites, socioeconomic circumstances, community attitudes, values, motivations and expectations need to be taken into account to ensure there is enough carrying capacity for the planned translocation.

### 4.4 Recommendations

Best practices for conservation translocations regarding genetic considerations are highly dependent on the context of the translocation. It must be noted that we distinguish two different components of genetic variation, encompassing different scales: (i) genetic clades, measured between populations, and (ii) heterozygosity, measured within populations or individuals. Our recommendations in this study focus mainly on the first component, expressed by the association of each LCU to a specific genetic clade. Therefore, we largely disregard within‐country translocations, even though these conservation interventions are fairly frequent, mostly as a response to human‐wildlife conflict. However, we do highlight those countries where within‐country translocations risk crossing boundaries between distinct genetic clades (see below). As mixing of genetic clades may have adverse effects, such as range‐wide homogenization and loss of adaptive potential, we urge managers to acknowledge differentiation between populations in their considerations of suitable source and target populations. Simultaneously, we acknowledge that, especially in the context of a reintroduction, i.e., a small initial population, within‐population diversity should be monitored and consecutive augmentation may be necessary to ensure that heterozygosity levels do not drop in subsequent generations.

As translocations may be approached as mimicking natural gene flow, in general, translocations with source/target combinations from close proximity are favoured. As a rough guideline for acceptable translocation distance from a genetics perspective, we propose to use a distance, which can reasonably be covered by natural, possibly multigenerational, dispersal. Natural dispersal of lions depends on a range of factors, including geography, landscape use, prey density and climate, males especially being capable dispersers, covering up to 200 km (Curry et al., 2015; Elliot et al., 2014; Packer & Pusey, 1987; Tumenta et al., 2013; Tuqa et al., 2014; Van Hooft et al., 2018). A stepping stone mode of dispersal over multiple generations therefore allows them to cover great distances. The naturally high dispersal capacity of males may be an argument for favouring male candidates in augmentation efforts; furthermore, they can spread their genes more efficiently in the target population by siring offspring with multiple females. However, their dispersal behaviour may simultaneously increase the risk of them leaving the landscape they have been translocated to. Site fidelity and possible attempts of the translocated individual to return to its original home range are particularly relevant when translocating damage causing animals (Boast et al., 2016), and therefore, some agencies use a minimum distance to reduce chances of the translocated animal returning (e.g., Kenya Wildlife Service uses 100 km). In addition, behavioural aspects, such as risks of disrupting existing pride structure and infanticide, must be taken into account. Survival of translocated individuals is a major concern when considering translocations as a conservation measure. For lions, this is mostly determined by conflict with local humans or resident lions (Morapedi et al., 2021).

In addition to isolation by distance, the current distribution of genetic variation is largely determined by barriers to dispersal. Spanning longer geographic distance and/or existing barriers for dispersal for augmentation of resident lion populations, or reintroduction with the potential for future connectivity, a translocation may result in mixing of genetic clades. This is associated with the risk of decreased fitness in offspring, known as outbreeding depression (Banes et al., 2016; Maheshwari & Barbash, 2011). Risks of outbreeding depression increase with genetic, geographical and environmental distances, as they may disrupt patterns of local adaptation (Edmands, 2007; Frankham et al., 2017). When monitoring for adverse fitness affects following a translocation, it is important to note that due to the temporary effect of heterosis (i.e., enhanced fitness observed in hybrid offspring) and increasing genetic incompatibilities as a result of genetic recombination, the onset of outbreeding depression may be delayed until the second or third generation of hybrid offspring (Bell et al., 2019; Goldberg et al., 2005; Whitlock et al., 2013). However, in general, the risk of outbreeding depression may be relatively low in comparison with inbreeding depression when dealing with small and isolated populations (Edmands, 2007; Frankham, 2015).

Reports of outbreeding depression in wildlife are still scarce (Huff et al., 2011; Marr et al., 2002; Marshall & Spalton, 2000), and local adaptation in lions has not been studied in detail. However, several papers show that it can be detected in wide‐ranging species with high mobility (Dures et al., 2019; Liu et al., 2018; Zhang et al., 2014; Zhao et al., 2013). Several studies have provided a framework on how to deal with local adaptation and integrate it in population vulnerability assessments (Flanagan et al., 2018; Razgour et al., 2019). Although these data are still largely lacking for lions, local adaptation should not be entirely disregarded as it may contribute to the formation of ‘eco‐types’, even on a relatively small geographic scale. Local adaptation has been addressed by two studies focussed on Botswana lions, which inhabit strongly differentiated habitats in relative close proximity, covering both arid desert habitat and wetlands (Dures et al., 2019; Moore et al., 2016). These studies did not include explicit tests for genomic adaptation; however, they showed that the existing population structure was best explained by taking environmental components into account (Dures et al., 2019). Although lions are known to be resilient and occur in a wide range of habitats, it is advisable to take ecological parameters into consideration when selecting a source/target population for translocation. This is particularly relevant for populations that are relatively unknown, both for their genetic background and their history, such as the lone lion in Gabon (Hedwig et al., 2018). A genetic sample from this individual was compared with mitogenome data from two historical samples from the same area, all showing a close genetic relationship to the Southwest haplogroup (Barnett et al., 2018). This example showcases the importance of such genetic assessments, as the nearest extant lion population from Gabon is located in Cameroon, despite it being a different subspecies. As a result, the planned population augmentation in Gabon, which aims to restore a breeding lion population in the Batéké landscape, will source lions harbouring the South West haplotype. Insights into local ecology can be used to further narrow down a suitable source population.

Another population for which such genetic considerations are a relevant argument is the Indian population. Although it is nested within the West and Central Africa clade, and therefore part of the subspecies *P. l*. *leo* (contrary to the former taxonomy, in which it was regarded as a separate subspecies *P. l. persica*), we advise against translocations from African lions to India or vice versa. The only situation in which a translocation of African *P. l*. *leo* individuals into India should be considered as if the Indian population has entered the extinction vortex (i.e., clear signs of inbreeding depression), and genetic rescue is needed to safeguard survival of the population. Despite the Indian population being very low in diversity as a result of multiple bottlenecks (Bertola et al., 2015; Driscoll et al., 2002), so far, reduced fitness due to inbreeding depression has not been clearly documented. However, a single population with low genetic diversity is particularly vulnerable and high mortality rates linked to canine distemper virus (CDV), babesiosis and other diseases have been reported on a regular basis for the Indian lion population (Anonymous, 2018; Kukreti, 2020; Sharma, 2020). From a management perspective, it would be advisable to translocate individuals from the Gir forest to suitable areas in the vicinity and manage it as a metapopulation. This approach would serve as a safety measure to contain a potential future disease and conserve the genetic background of Indian lions.

Since the CITES database only covers international trade, the presented data do not give insight into within‐country translocations, even though several countries harbour multiple lion clades (Figure 1). In these cases, namely Nigeria, DRC, Kenya, Namibia, Zambia, Zimbabwe, Botswana and possibly Uganda, within‐country translocations should also carefully consider suitable source and target populations. For several countries, a suture zone exists in which multiple clades co‐occur and natural hybridization has been described for some populations, notably in Ethiopia (suture zone 1, likely extended into Sudan and South Sudan) and Zambia (suture zone 2, likely extended into Malawi and Mozambique). In the case of suture zone 1, this region represents two subspecies of lions. In the event of a translocation involving these suture zones, we suggest sourcing individuals from the same suture zone. Due to the dire situation facing the northern subspecies *P. l*. *leo*, we also suggest prioritizing *P. l. leo* individuals in the case of the *P. l. leo*/*P. l. melanochaita* suture zone (suture zone 1), assuming sustainable off‐take from a source population is possible. Although we focus our assessment on continent‐wide patterns of diversity, population structure can also be determined on a more local scale. Few countries have had a country‐wide or regional assessment of lion population structure so far, but if this information is available, e.g., for Tanzania (Smitz et al., 2018) or Zambia (Curry et al., 2015), we urge managers to include this information to make informed management decisions.

Beyond the impact on lion populations, undocumented translocations can impede legal actions against the trade in both live lions and lion body parts. Successful prosecution for such illegal trade may require evidence of their likely origin, which is commonly determined using genetic methods to assign an evidence item to a particular population while excluding it from others. This requires the population of origin to be sufficiently genetically distinct from other populations (Ogden & Linacre, 2015). A tool to provide such information for law enforcement intelligence is being made available for lions (lionlocalizer.org). However, the presence of hybrid individuals resulting from translocations may introduce doubt into the genetic origin of a lion or lion body part being used as evidence, thereby hindering the ability to utilize geographic assignment testing for forensic purposes. Documentation of management interventions will be helpful for understanding future patterns of diversity and possible forensic applications.

As we move towards the future, with ongoing land conversion and possible future local extinctions, we need to maintain a flexible attitude and be willing to reconsider our recommendations as new data become available and the situation on the ground may change. Making decisions on a case‐by‐case basis, including a wider context of both source and target populations, will contribute towards maintaining diversity. We emphasize that this paper is purely on the genetic aspect of translocations, and we acknowledge that other considerations must be taken into account. Several resources are available for dealing with translocations in general, such as the IUCN Translocation Guidelines (IUCN, 2013), or specifically for lions, such as the Guidelines for the Conservation of the Lion in Africa (IUCN SSC Cat Specialist Group, 2018), the guidelines produced by the African Lion Working Group (African Lion Working Group, 2016), and a previous assessment on the situation in South Africa (Slotow & Hunter, 2009). Acknowledging the complexity of these management interventions, we hope that this paper serves as a starting point, supporting wildlife managers in including the genetic component in their decision‐making process, which ensures that the genetic variation, and therefore the evolutionary potential, of the lion remains intact.

### 4.5 Concluding remarks

The genetic variation present across the natural range of a species serves as its evolutionary potential to adapt to changes in its environment, such as climatic shifts, epidemics etc., thereby making it more resilient. If this variation declines, e.g., by local extinctions or by uncontrolled moving of individuals during translocations, the adaptability and the chances for long‐term survival of the species in the wild are impacted. We therefore urge managers to take genetic information, such as assignment to a genetic clade, into account when selecting source/target populations for conservation translocations. We present the current knowledge on the distribution of genetic variation in the lion in a comprehensive overview, hoping that this will support managers to integrate these insights when making decisions regarding source/target populations for translocations. This paper is not meant to fuel a debate on the feasibility of conservation interventions; rather, our hierarchical approach reflects our aim to deliver a tool, which includes the idealism‐pragmatism spectrum. Although this paper uses the lion as a case study, this approach is more broadly applicable. Co‐distributed species often show similarity in their phylogeographic patterns, depending on their ecology and demographic history, and hence may benefit from similar recommendations as are represented here for the lion.

## CONFLICT OF INTEREST

The authors declare that there is no conflict of interest.

## Supporting information

Appendix S1

Table S1‐S6

## Data Availability

Data for this study are available in the Supplementary Material. No new genetic data were generated for this study.
