# Supplementary material for: Genetic guidelines for translocations: Maintaining intraspecific diversity in the lion (Panthera leo)
Source: Evol Appl. 2021 Dec 6;15(1):22–39. doi: 10.1111/eva.13318 (PMC8792481; doi:10.1111/eva.13318)
Supplement: Supplementary file 1 — Appendix S1 [file EVA-15-22-s002.docx]

**Appendix S1**

**Details on the use of the CITES Trade Database**

When mining the import and export permits for lion (*Panthera leo*) transactions, the data were restricted to transactions of live lions amongst African countries, and the import of live captive lions into lion range states. Additional sources of information for South Africa were obtained from (i) South Africa’s unpublished annual reports submitted to the CITES Secretariat for the period 2008—2015 [(Williams et al. 2017b)](https://paperpile.com/c/hqZtkX/fJrZH); (ii) published evidence of lion translocations between countries [(Slotow and Hunter 2009; Briers-Louw, Verschueren, and Leslie 2019; African_Parks 2015)](https://paperpile.com/c/hqZtkX/ZODg+Q5e0+gS7M); (iii) unpublished data from two surveys of South African captive lion keepers conducted by V.L. Williams (one of which is referred to in [(Williams and ’t Sas-Rolfes 2019)](https://paperpile.com/c/hqZtkX/ab6Y7); (iv) unpublished data, (S.M. Miller; J. Selier pers. comm.). These additional data sources allowed some transactions on the CITES Trade Database to be crosschecked and appraised, in particular for corroborating the transaction purposes (e.g. reintroduction or commercial), sources (e.g. wild or captive), and numbers of translocated individuals listed on the permits. It needs to be noted that these numbers reflect the intention of trade, and the maximum number of live lions that were permitted to be exported/imported by permit Issuing Authorities, and may therefore be an overestimate of the true number of traded individuals.

Transactions must specify the original sources of the lions, which are designated by the following CITES codes: (i) W — specimens taken from the wild; (ii) C — animals bred in captivity in accordance with Res. Conf. 10.16 (Rev.); (iii) F — animals born in captivity (F1 or subsequent generations) that do not fulfil the definition of ‘bred in captivity’ in Res. Conf. 10.16 (Rev.); and (iv) R — specimens reared (‘ranched’) in a controlled environment, or juveniles from the wild, where they would otherwise have had a very low probability of surviving to adulthood  (CITES, 2013). Here, we consider individuals from category ‘W’, ´F´ and ‘R’ as wild, as we focus on the genetic origin of the animals, rather than the conditions in which they live (but see the introduction of category ‘W2’ in Supplemental Table 5).

Trade of live lions across Africa are assigned on the CITES Trade Database to 10 different purpose codes. Four of these purpose codes were selected as relevant in the context of this study, i.e. moderate to high risk of translocated individuals breeding with a wild, resident lion population (defined in [(CITES 2007b, 2013)](https://paperpile.com/c/hqZtkX/jUXui+FcSlP):

(i) N – *reintroduction or introduction in the wild*; defined as live specimens to be used in conservation efforts to reintroduce or supplement wild populations, including the movement of specimens for non-commercial breeding in captivity or artificial propagation in support of *in situ* conservation efforts

(ii) T — *commercial*; defined as trade in live specimens for economic benefit not covered under B (Breeding in captivity), M (Medical, including biomedical research) or Z (Zoo)

(iii) B — *breeding in captivity*; defined as trade in live specimens for breeding in captivity, including the production of progeny, for economic benefit

(iv) H — *hunting trophy*; defined as personal sport-hunted trophies transported by the hunter or their legal representative. As trophy transactions are usually for the derivatives of the hunted animal and not live lions, we presume that in these cases live animals were translocated for the purposes of releasing them for hunting, which means they may have had an opportunity to breed if circumstances permitted.

Here, we make the distinction between lion for (re)introduction purposes (‘N’) and lions traded for other purposes, but with the risk of interbreeding with free roaming lions at the target area (‘T’, ‘B’ and ‘H’). We included the latter category as well as and captive-sourced, since (a) anecdotal evidence suggests that individuals translocated for these purposes might occasionally have opportunities to integrate into resident wild lion populations (e.g. lions from South African captive breeding facilities; VL Williams, unpublished data), and (b) South Africa inconsistently assigned codes ‘C’ and ‘W’ to free-roaming lions up to 2012 [(Williams et al. 2017a)](https://paperpile.com/c/hqZtkX/U3kC3), including those from small fenced reserves in the managed metapopulation. Exporter and importer CITES permits occasionally list inconsistent sources; for example, lions translocated to Rwanda in 2015 originated from the Phinda Private Game Reserve and Tembe Elephant Park (African_Parks, 2015), and the exporter and importer permits classified them as ‘C’ and ‘W’ respectively. We thus applied the following rules when extracting information from the CITES Trade Database: (i) inconsistent sources are classified according to the exporter permit (N=86 individuals); (ii) inconsistent purposes are classified according to the importer permit; (iii) unlisted sources (N=99 individuals) are classified as ‘C’ (unless otherwise specified, Suppl. Table 4); and (iv) used the largest of two inconsistent quantities listed by an exporter/importer, to reflect the maximum number intended for translocation. Some of these could be resolved with additional information, as is explained in Supplemental Table 5.

**Limitations CITES Trade Database**

There are limitations to the accuracy of the information available on the database based on: (1) *the* *basis of the reporting and compilation*; the majority of annual reports submitted do not specify whether they were compiled based on quantities listed on permits issued or actual numbers of traded specimens [(CITES 2013, [a] 2007; Sinovas et al. 2016)](https://paperpile.com/c/hqZtkX/FcSlP+PpA9n+vJ6Tc) – hence trade in live lions is overestimated for countries submitting reports based on the permits issued; (2) *timely submission of annual reports to the CITES Secretariat*; there are gaps in the annual reports for some countries like Namibia, Malawi, Tanzania and Zambia [(Sinovas et al. 2016)](https://paperpile.com/c/hqZtkX/vJ6Tc), and delayed submissions by other countries, resulting in the most up-to-date records not being available – hence trade is under-estimated for countries that do not submit reports; (3) *completeness and quality of the annual reports*; not all annual transactions are reported [(Chandran, Krishnan, and Nguyen 2011; CITES 2007a, 2013; D’Cruze and Macdonald 2016)](https://paperpile.com/c/hqZtkX/FMeV5+PpA9n+FcSlP+DKYXt) – hence trade is under-estimated in countries that submit poor and incomplete information; and, (4) *assignment of the correct purpose and source codes*; incorrect or inconsistent codes are sometimes assigned to the transactions (e.g. reintroduction or commercial, wild or captive) [(CITES 2007a; Williams et al. 2017a, 2015)](https://paperpile.com/c/hqZtkX/PpA9n+U3kC3+7ewS7), potentially deliberately on occasion – thereby obfuscating the quantitative and qualitative accuracy on transboundary movements of lions and the relative purposes thereof. As a result, the available annual trade data are not accurate for all Parties concerned. Accordingly, and with respect to transboundary exports/imports of live lions, it is reasonable to presume (i) that intra-African compliance with CITES regulations has been inconsistent, (ii) that incorrect assignment of the correct purpose code to some transactions has resulted in an under-estimate of lions that were exported for the specific purposes of (re)introducing them into the wild; (iii) that there have been over-estimates of the number of lions exported/imported by some countries that are compliant with CITES regulations, and (iv) yet, under-estimates of lions for countries that are non-compliant and/or inaccurate in their annual reporting. Apart from the points mentioned above, the recorded numbers based on CITES permits do not cover any incidences of intra-African illegal trade, which cannot be excluded in the context of lion translocations.

**References**[African_Parks. 2015. “African Parks to Translocate and Reintroduce Lions into Akagera National Park, Rwanda.” *African Parks*.](http://paperpile.com/b/hqZtkX/gS7M)<https://www.africanparks.org/newsroom/press-releases/african-parks-translocate-reintroduce-lions-into-akagera-national-park-rwanda>[.](http://paperpile.com/b/hqZtkX/gS7M)

[Briers-Louw, Willem D., Stijn Verschueren, and Alison J. Leslie. 2019. “Big Cats Return to Majete Wildlife Reserve, Malawi: Evaluating Reintroduction Success.” *African Journal of Wildlife Research* 49 (1): 34–50.](http://paperpile.com/b/hqZtkX/Q5e0)

[Chandran, Remi, Padmanabhan Krishnan, and Khoi Nguyen. 2011. “Wildlife Enforcement Monitoring System (WEMS): A Solution to Support Compliance of Multilateral Environmental Agreements.” *Government Information Quarterly* 28 (2): 231–38.](http://paperpile.com/b/hqZtkX/FMeV5)

[CITES. 2007a. “Interpretation and Implementation of the Convention. Compliance and Enforcement Issues. CoP14 Doc. 26 (Rev. 1).”](http://paperpile.com/b/hqZtkX/PpA9n)

CITES[. 2007b. “Interpretation and Implementation of the Convention - Trade and Control Marking Issues CoP14 Doc. 39.”](http://paperpile.com/b/hqZtkX/jUXui) <https://www.cites.org/sites/default/files/eng/cop/14/doc/E14-39.pdf>[.](http://paperpile.com/b/hqZtkX/jUXui)

CITES[. 2013. “A Guide to Using the CITES Trade Database. Version 8.”](http://paperpile.com/b/hqZtkX/FcSlP) <https://trade.cites.org/cites_trade_guidelines/en-CITES_Trade_Database_Guide.pdf>[.](http://paperpile.com/b/hqZtkX/FcSlP)

[D’Cruze, Neil, and David W. Macdonald. 2016. “A Review of Global Trends in CITES Live Wildlife Confiscations.” *Nature Conservation*. https://doi.org/](http://paperpile.com/b/hqZtkX/DKYXt)[10.3897/natureconservation.15.10005](http://dx.doi.org/10.3897/natureconservation.15.10005)[.](http://paperpile.com/b/hqZtkX/DKYXt)

[Sinovas, P., B. Price, E. King, F. Davis, A. Hinsley, A. Pavitt, and Others. 2016. “Southern Africa’s Wildlife Trade: An Analysis of CITES Trade in SADC Countries.” Technical report prepared for the South African National Biodiversity ….](http://paperpile.com/b/hqZtkX/vJ6Tc)

[Slotow, Rob, and Luke T. B. Hunter. 2009. “Reintroduction Decisions Taken at the Incorrect Social Scale Devalue Their Conservation Contribution: The African Lion in South Africa.” *Reintroduction of Top-Order Predators*, no. 5: 43.](http://paperpile.com/b/hqZtkX/ZODg)

[Williams, Vivienne L., Andrew J. Loveridge, David J. Newton, and David W. Macdonald. 2017a. “A Roaring Trade? The Legal Trade in Panthera Leo Bones from Africa to East-Southeast Asia.” *PloS One* 12 (10): e0185996.](http://paperpile.com/b/hqZtkX/U3kC3)

[Williams, Vivienne L., Andrew J. Loveridge, David J. Newton, and David W. Macdonald](http://paperpile.com/b/hqZtkX/U3kC3)[. 2017b. “Questionnaire Survey of the Pan-African Trade in Lion Body Parts.” *PloS One* 12 (10): e0187060.](http://paperpile.com/b/hqZtkX/fJrZH)

[Williams, Vivienne L., D. J. Newton, Andrew J. Loveridge, and David W. Macdonald. 2015. “Bones of Contention: An Assessment of the South African Trade in African Lion Panthera Leo Bones and Other Body Parts.” *TRAFFIC & WildCRU Joint Report*.](http://paperpile.com/b/hqZtkX/7ewS7)

[Williams, Vivienne L., and Michael J. ’t Sas-Rolfes. 2019. “Born Captive: A Survey of the Lion Breeding, Keeping and Hunting Industries in South Africa.” *PloS One* 14 (5): e0217409.](http://paperpile.com/b/hqZtkX/ab6Y7)
